# Operational challenges to continuous LLIN distribution: a qualitative rapid assessment in four countries

**DOI:** 10.1186/s12936-016-1184-y

**Published:** 2016-03-01

**Authors:** Katherine Theiss-Nyland, Waqo Ejersa, Corine Karema, Diakalia Koné, Hannah Koenker, Yves Cyaka, Matthew Lynch, Jayne Webster, Jo Lines

**Affiliations:** Infectious Disease Epidemiology Department, London School of Hygiene and Tropical Medicine, Keppel Street, London, UK; Malaria Control Unit, Ministry of Health, Nairobi, Kenya; Malaria and other Parasitic Diseases Division, Rwanda Biomedical Centre, Kigali, Rwanda; Programme National de Lutte Contre le Paludisme, Ministere de la Sante et l’Hygiene Publique, Bamako, Mali; Center for Communication Programs, Johns Hopkins Bloomberg School of Public Health, Baltimore, MD USA; Tropical Health LLP, London, UK; Disease Control Department, London School of Hygiene and Tropical Medicine, Keppel Street, London, UK

**Keywords:** Continuous distribution, LLINs, Routine distribution, Monitoring and evaluation, Rapid assessment process, ANC, EPI

## Abstract

**Background:**

The World Health Organization recommends that long-lasting insecticidal nets (LLINs) for malaria prevention should be distributed continuously through antenatal care (ANC) and the expanded programme on immunization (EPI) in addition to mass campaigns. Despite these recommendations, the continuous distribution (CD) of LLIN distribution through ANC and EPI is not policy in many countries, and where there is a policy, implementation is incomplete. This study aims to identify the operational strengths and weaknesses of LLINs CD in four country programmes in sub-Saharan Africa.

**Methods:**

A qualitative rapid assessment process was conducted using semi-structured individual and group interviews at the national, sub-national, and facility level in four countries. Seventy participants were included (23 in Kenya, 13 in Malawi, 18 in Mali and 16 in Rwanda), drawn from malaria programmes, ANC and EPI programmes, government logistics units, and partner organizations. Interviews were structured to identify themes within a health systems approach. Policy and guideline documents and data collection tools were reviewed as a means of triangulation. Data analysis focused on pre-determined and emergent themes.

**Results:**

The four countries used a wide variety of management systems for the supply of LLINs to routine services. Issues related to quantification, supply logistics and data collection all contributed to stock-outs at facility level. None of the four countries had guidelines for responding to stock-outs or system enabling local staff to request additional supplies of LLINs. In all four countries, data collection of LLIN distribution was incomplete or absent at facility level, and such data were not used for planning. Training of staff at the facility level was implemented less frequently than national and sub-national staff would have preferred. Logistics systems, independent of other commodities, and in-country partner support strengthened the continuous distribution of LLINs.

**Conclusions:**

In these countries, stock-outs were the most important single obstacle to the smooth operations of continuous LLIN distribution. Stock-outs can be avoided if facilities have the capacity to place orders for LLIN resupply as needed. Revised data collection and management systems for LLIN distribution have the potential to increase coverage of the target populations by improving LLIN stock-out response, and strengthening monitoring and evaluation of distribution.

**Electronic supplementary material:**

The online version of this article (doi:10.1186/s12936-016-1184-y) contains supplementary material, which is available to authorized users.

## Background

Long-lasting insecticidal nets (LLINs) are widely promoted for malaria prevention, and are distributed free of charge in 88 countries [1]. LLINs have been distributed primarily via large mass campaigns, following the success of the first national LLIN distribution campaign in Togo in 2004 [2], and thanks to funding from the Global Fund [3]. In 2007, the World Health Organization (WHO) began recommending both universal coverage campaigns and the continuous distribution of LLINs: providing LLINs to pregnant woman and infants through routine ANC and EPI services [4]. More explicit recommendations were later released, which further stressed the need for continuous distribution, and noted that, while campaigns were the most efficient method for rapidly scaling up LLIN ownership, a method which ensured a consistent stream of new nets entered communities, to maintain coverage between mass campaigns, was necessary [5–7]. “Giving higher priority to routine services, such as ante-natal clinics (ANC) and the expanded programme on immunization (EPI) as a means of LLIN distribution to sustain Universal Coverage” was encouraged [6]. The WHO recommendation further states that, “Continuous distribution channels should be functional before, during, and after the mass distribution campaigns to avoid any gap in universal access to LLINs [5].”

In practice, there has been limited implementations of LLIN continuous distribution through ANC, and even less through EPI. Globally, 49 countries distribute LLINs through ANC, and 29 do so through EPI [1]. For those countries with continuous distribution, the 2013 World Malaria Report stated that in a three year period nets were only available for 55 % of women attending ANC, and 34 % of children attending EPI [1].

Compared to LLIN campaigns, there has been less research on continuous LLIN distribution through ANC and EPI, and this research has tended to focus on the expected coverage which could be achieved, the feasibility of implementation, or the cost per net delivered [8–15]. Few studies have looked at the performance of these delivery systems at reaching their target groups, and the factors that influence the coverage that is actually achieved [16, 17].

The aim of this study was to examine the operational systems used to distribute LLINs through ANC and EPI in four African countries, as seen by the professional staff who manage and support the process, and to identify the strengths and weaknesses of these systems and the main operational barriers to better performance.

## Methods

A qualitative Rapid Assessment Process (RAP) was conducted in Kenya, Malawi, Mali, and Rwanda between March and May of 2014. The RAP method uses iterative semi-structured interviews and triangulation to provide qualitative evidence for policy makers and programme planners in a limited time-frame [18]. The countries for this study were selected from 20 President’s Malaria Initiative (PMI)-supported countries to include countries from both anglophone and francophone Africa, with a range of malaria transmission settings, a variety of continuous LLIN distribution experiences and policies, and a range of levels of coverage of LLIN, ANC and EPI services.

Two to four health facilities were selected in each country by the national malaria programme using purposive sampling, with input from local partner organizations. Purposive sampling was used to generate a “typical case sample” whereby the facilities in each country would represent the normal or average service delivery, and could be compared across countries in the study [19]. Facilities were eligible for selection by the national malaria programme if they were non-urban, away from major roads, accessible from the capital within one day via car, in a malaria-endemic area, seen to be “average performing” in terms of malaria/LLIN delivery and general services, and the lowest level of health service delivery providing community health, maternity and EPI services (Kenya: Health Centres—level 3; Malawi: Health Centre; Mali: CSCOM; and Rwanda: Health Centre). In Kenya, four facilities were selected in Western and Nyanza provinces. In Mali, two facilities were selected in Koulikoro Region. In Malawi, one facility was selected in each of the three regions. Two Malawian facilities were government clinics, and one was supported by the Christian Health Association of Malawi (CHAM). One government facility originally selected was replaced due to reported heavy work-load of facility staff—the time available was not enough to wait until the end of clinic hours, and interrupting health services was not considered appropriate. In Rwanda, three facilities from the North and South were included in the study. In Rwanda, one of two originally selected facilities was unreachable due to heavy rains and flooding. As an alternative, two facility heads from two other facilities, who were at a nearby regional meeting, were opportunistically interviewed together.

At the national, sub-national, and facility levels, interviewees were purposively selected according to their role in the management or implementation of continuous LLIN distribution, ANC services, and/or EPI services. In total 38 interviews were conducted with 70 participants (Table 1). Semi-structured interviews were conducted with individuals or in small groups, depending on the availability and preference of interviewees. Interviews focused on the continuous distribution of LLINs through routine health services. In order to ensure that key health systems issues were covered, the interview guide was structured around the WHO “6 building blocks to health system strengthening”: Service delivery; health workforce; information; medical products, vaccines, and technology; financing; and leadership and governance [20]. The interview guides also included questions on ANC and EPI product logistics, and on the most recent mass LLIN distribution campaign and vaccination campaigns. Respondents were not asked to directly compare the logistics of different products. Probing questions and questions-of-clarification were used iteratively to explore key themes that arose, or when more information was required (see national and facility level interview guides in Additional files 1 and 2). In addition to the interviews, policy and guideline documents were reviewed at the national level, and data collection tools and reporting forms at health facilities.

**Table 1:** Interviews (participants) included by category, by country

	Mali	Malawi	Kenya	Rwanda	Total
Facility	2 (9)	3 (3)	4 (6)	2 (5)	*11 (23)*
Sub-national health office	1 (4)	1 (2)	1 (7)	2 (3)	*5 (16)*
National malaria control unit	2 (2)	1 (2)	1 (6)	2 (2)	*6 (12)*
National reproductive health/MCH Unit	1 (1)	1 (1)	1 (1)	0.5^a^ (1)	*3.5 (4)*
National EPI Unit	–	1 (1)	1 (1)	0.5* (1)	*2.5 (3)*
Logistics (national level)	–	1 (1)	–	3 (3)	*4 (4)*
Partner organizations (national level)	1 (2)	3 (3)	1 (2)	1 (1)	*6 (8)*
*Total*	*7 (18)*	*11 (13)*	*9 (23)*	*11 (16)*	*38 (70)*

^a^One interview covering both reproductive health and EPI in Rwanda

Interviews were conducted by two of the authors (KTN and YC), using English in Malawi and Kenya, French in Mali, and a combination of French and English in Rwanda. Before each interview began, the aims of the project were explained, information sheets were provided to each participant and written consent was obtained. Each interview was led by one team member, with the other team member taking notes and probing where further information was needed. All interviews were recorded, translated if in French, transcribed and entered into Nvivo10 for data management and analysis. The data were analysed by KT, starting with the previously-identified themes. The analysis was then further developed to explore additional emergent themes that were considered important for understanding the operational barriers to LLIN continuous distribution.

The protocol was reviewed and approved by the London School of Hygiene and Tropical Medicine (LSHTM) and the Johns Hopkins Bloomberg School of Public Health ethics review boards. In each country the national malaria control programme provided the research team with a letter of approval, and supported the study as part of a routine programme evaluation.

## Results

**Fig. 1:**
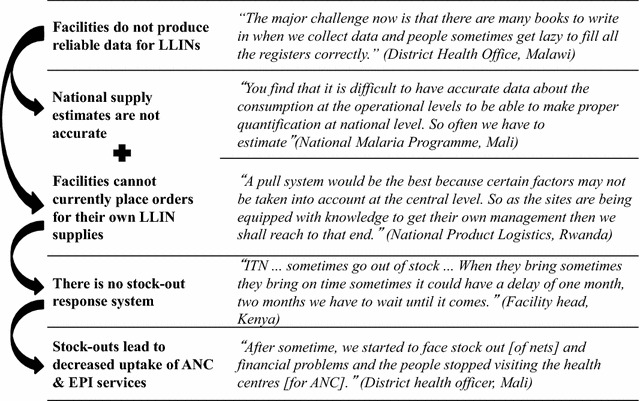
Operational barriers leading to stock-outs and decreased confidence in services

Based on the perceptions of the respondents, five thematic areas emerged as central in terms of operational barriers to continuous LLIN distribution in all four countries: (1) Quantification; (2) Logistics systems; (3) Stock-outs; (4) Training; and (5) Data management. These replaced the a priori themes of policies and management; logistics; programme implementation and human resources; and data collection, management and use. Logistics and data management are both a priori and emergent themes, while stock-outs, training and quantification emerged as more narrowly focused topic areas of importance. These five thematic areas captured the weaknesses and challenges identified by respondents in all four countries. A summary of the findings across countries, by these five thematic areas, is presented in Table 2. Figure 1 describes the operational barriers, following the LLIN distribution path, as a cascade leading to stock-outs at the facility level.

**Table 2:** Summary of Findings, by thematic area

Thematic area	Specific area	Key findings
Quantification		-All programmes conduct annual quantification exercises to produce supply needs -Both population estimates as well as facility consumption data are used to produce LLIN quantifications -Poor data quality at the facility level results in national-level estimations that may have errors
Logistics systems	Distribution management	-LLIN distribution managed separately from other commodities -Ideal scenario is integrated distribution; practical solution is separate -Bulk of nets was not identified as a major challenge for distribution -Heavily led by partner organizations
	Supply and restock	-Each country had a different frequency of restock -Commodities with dedicated funding and distribution most reliable supply chain (e.g. HIV, EPI, LLIN) -Order placed primarily top-down “push” not bottom-up
Stock-outs	Occurrence	-All countries had reported stock-outs by facilities
	Remedy	-Make-shift stock-out corrections -No clear stock-out guidelines in any country
Training		-Lack of funding available -Focused on new staff
Data management	Collection	-Overwhelming amount of registers and report forms for health workers to fill out at the facility level -Missing data were common in registers that were reviewed -Special LLIN distribution register produced by partner organizations
	Use	-Facilities rarely used data for progress tracking -National and sub-national programme staff used facility reported data -National malaria programme conducted surveys in addition to routine data to track programme impact

### Quantification

In each country, the malaria control unit used population statistics (estimations of expected pregnancies and births) and facility consumption data (LLINs distributed to/from each facility) to produce a “quantification”—a plan for the quantity of LLINs needed in the next year. The quantification process in all four countries was managed at the national level with support from partner organizations, some input from sub-national level staff, but no direct input from facility staff. The national malaria programme managers in Kenya and Mali reported using primarily population statistics to estimate the LLINs needed, due to concerns about consumption data quality and completeness.

*“A population grows, so we cannot use consumption data to go and say that this is now what is expected.” (National Malaria Programme, Kenya)*

*“You find that it is difficult to have accurate data about the consumption at the operational levels to be able to make proper quantification at national level. So often we have to estimate… For example, we consider the percentage of pregnant women in the population and based on that we estimate the number of nets.” (National Malaria Programme, Mali)*

In Malawi and Rwanda, LLIN quantifications also used population estimates as the primary data source, but these estimates were adjusted using consumption data reported from facilities.

*“Every quantification is based on all the methods so regardless you have 100* *% or 0* *% reporting you look at your population, you need to locate your consumption if you have any numbers. So any quantification cannot be based on one method only, you have to work with all the methodologies, provided you have the data” (Partner organization, Malawi)*

### Logistics systems

In all four countries, at the national level, the logistics system (the organization, management, and implementation of storage, transport and distribution) for routine LLINs was managed separately from that of other health service commodities with support from partner organizations. Health systems with dedicated funding and/or independent distribution, such as HIV services, LLINs, and vaccines, were identified by facility staff in the four countries as having the most reliable supply chains. Conversely, at the national and sub-national levels, and within partner organizations, staff felt that ideally LLIN would be managed and distributed by the government, as part of an integrated supply system for health commodities. However, given the resources and capacity currently available, and the experience and expertise brought by partner organizations, both national level government and partner officials stated that at the current time separate systems were more functional.

*“At the moment we… are running a parallel supply chain. In other words we have got a parallel system where we do storage and distribution. In an ideal situation all storage and distribution will go through the national system but in the country there are several similar supply chains” (Partner Organization, Malawi)*

Storage space for LLINs at the national level, directly after shipments were received from suppliers, was presented as a problem when international shipments came in large amounts.

*“Sometimes the nets come at once and we find ourselves running up and down to find warehouses to stock them… Keeping nets for a long period does not happen often but in the past we have experienced situation where the number of nets was higher than what our storage capacity could accommodate.” (National Health Product Logistics, Rwanda)*

**Fig. 2:**
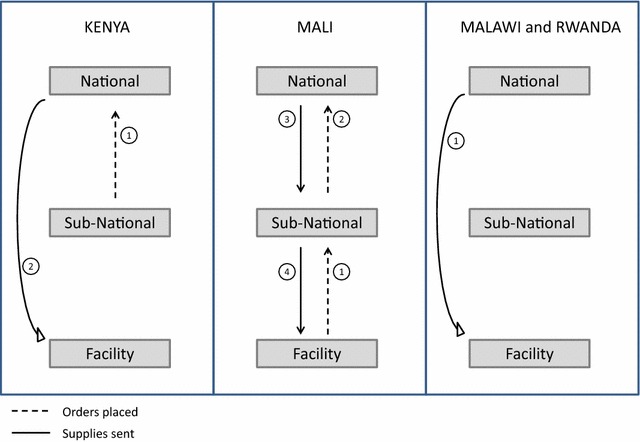
Supply order and fill process in each country. *Kenya* (*1*) Sub-county health office places order to national programme on behalf of facilities, based on sub-country quantification developed by the sub-county health office with support from partners; (*2*) national programme fills order to facilities from regional storage warehouses. *Mali* (*1*) Facilities place request order to district based on consumption; (*2*) district collates all facility request, and places request to national programme; (*3*) national programme “corrects” order based on LLIN availability and its own quantification estimates and fills order to the district; (*4*) district adjusts and fills facility orders based on available supplies. Malawi and Rwanda: (*1*) Facilities supplied based on national distribution plan; Malawi uses regional storage space to keep LLIN supplies between national distributions

The frequency of resupply of LLINs to local facilities varied between countries. There was one supply of LLINs per year in Rwanda, two per year in Mali, and four per year in Kenya. In Malawi, facilities were re-supplied monthly. The resupply system was different in each country, illustrated in Fig. 2. With the exception of Mali, the LLIN supply chain largely skipped over the sub-national level, providing LLINs to facilities directly from the national level. The supply chain in Mali and Kenya included stock requests from lower levels, while Malawi and Rwanda provided LLINs to facilities based on national supply plans. In each country, however, the sub-national level was informed of the LLIN shipments being provided to facilities. No one system created a more consistent supply of LLINs at the facility level than any other.

In Rwanda and Malawi, health facility staff expressed concerns about the management of stock and of storage space for LLINs once LLIN stock reach the facility. In Kenya and Malawi, LLINs were usually stored in the same consulting room where ANC services were provided. In larger district facilities in Malawi, the main stock of LLINs was kept in the pharmacy stores, and smaller quantities were kept in service delivery rooms. In Rwanda and Mali, LLINs were kept in a storage space that was either dedicated to LLINs alone, or shared with a variety of other commodities—in Mali these facilities were kept locked due to LLIN security concerns. In facilities in all four countries, the storage location of LLINs seemed to be dictated by the available space, layout of the facility, and convenience for health workers. Although storage space was frequently cited as an inconvenience, it was never mentioned as a cause of service failure.

In all four countries, facilities were not able to place orders for LLIN supplies, and would, instead, wait for shipments from the national level.

*“We are able to know that in this year we are going to distribute so many nets in these health facilities. So from this, we are able to form itineraries that will be able to fit, and we are able to achieve the objective of distributing the nets… We have our work plans, based on the national Malaria guideline. Like, if these nets are required to be given to a certain facility, we are able to work within that and be able to deliver the nets within the stipulated time.” (Partner Organization, Kenya)*

In all four countries respondents at all levels expressed an interest in developing and/or strengthening a system whereby facilities can place orders for more LLINs, based on stock levels and need.

*“A pull system would be the best because certain factors may not be taken into account at the central level. So as the sites are being equipped with knowledge to get their own management then we shall reach to that end.” (National Health Product Logistics, Rwanda)*

In Kenya, the LLIN system was slowly transitioning to one in which lower levels submit orders based on need. This system had been rolled out and implemented down to the sub-county (sub-national level) at the time of the evaluation, and there were plans to extend it to facility level. In Malawi and Mali, the staff at the national level was less confident about facilities’ ability to order supplies for restock consistently and accurately.

*“Since [facilities] are not able to quantify properly the need for the nets, we rely on statistics and hypothesis based on the population.”(National Malaria Programme, Mali)*

### Stock-outs

In all four countries, staff at facilities described experiencing stock-outs with no formal system to rectify this. As a result, staff at facilities reported using informal methods with mixed outcomes. In Rwanda, LLIN stock-cards were used to track stock levels, which were reported to the district. At the district, the stock levels were entered in the electronic information system. When stock-outs occurred they were reported through this same system, and could be seen at the national level. In Malawi, facilities reported waiting for more supplies of LLINs during stock-outs, rather than actively seeking re-stock from national level, although in some cases, there was an informal stock-sharing system with facilities nearby when there were stock-outs. In Kenya, facilities reported having stock-outs generally lasting no more than 1 week before re-supply, with a maximum wait-time of 3 months. In Kenya, facilities would call the partner organization contact and/or sub-national malaria focal person directly to report stock-outs.

*“[ITN supply] has been quite irregular…When they come they have to be supplying a whole area, so sometimes it could take three months, four months; we can go even three months on end without supplies. So that one is completely out of our control.” (Health Facility, Kenya)*

*“Sometimes we face stock outs and we are not able to give nets for example now we do not have any so people go home without nets.” (Health Facility, Malawi)*

*“After sometime, we started to face stock out [of nets] and financial problems and the people stopped visiting the health centres [for ANC]…That is, to me, the only reason that negatively impacts the implementation of these policies.” (District Health Office, Mali)*

### Training

Staff at health facilities reported receiving training for continuous LLIN distribution less frequently than for other programmes and tasks. For example, staff described annual trainings on intermittent preventive treatment for malaria in pregnancy, and for vaccination services, in all countries. At national and sub-national levels, across all four countries, staff expressed concern that there was not enough funding available for the training that was planned and needed. National malaria programme staff and partner organizations reported limiting LLIN trainings to new staff, instead of including all staff, as a way to deal with limited funding and high staff turnover.

*“If we have funds from donors we organize trainings for the staff but for the past two years trainings have been through the [National] Malaria Control Programme. They provide training about malaria in general. We were supposed to have trainings about the ITN distribution … but we did not.” (District Health Office, Malawi)*

*“There is an issue of staff turnover. The staff you train this year is not the staff you find next year so there is always a need for more trainings.*.. *When someone is new, we have to train the person and we do not always have funds to do that.” (National Health Product Logistics, Rwanda)*

### Data management

Across countries, health workers at the facility level reported an overwhelming number of registers and reports to fill as part of the job, which captured all services delivered throughout the health centers. In all four countries, when viewing registers, there were examples of missing data, inaccurate entries and unorganized paperwork. In many cases a report format or tool was described in an interview but was unable to be located when requested. While many registers and reporting forms were official government forms, some additional report forms were produced by partner organizations for monitoring the programmes that they sponsored. In one facility visited in Kenya, the head of the facility listed 23 separate reports that were required every month.

*“The major challenge now is that there are many books to write in when we collect data and people sometimes get lazy to fill all the registers correctly.” (District Health Office, Malawi)*

*“A challenge often at facility level is making sure that these two registers speak to one another because at times you might find that one is fully filled, so because they know that [we] will use that net pack record, because that is the one we use for consumption data and so on, sometimes they forget to fill in the net details on the children CWC register and on the mother’s register as well.” (Partner Organization, Kenya)*

In Kenya, Malawi and Rwanda, electronic health information systems were available at the national level, and in some regions and districts, but not at the facility level. The number of LLINs distributed via ANC and EPI was included as an indicator in these systems in all three countries. Other indicators that were of interest to national level staff were stock shortages/stock-outs and shipments, though these data were not available in the systems at the time of the assessment. At the sub-national and national level in all countries, regardless of the presence of electronic record systems, there was distrust in the information being captured and produced at the health facility level. This was expressed in both countries that did and did not use facility consumption data for quantification. Sub-national staff reported contacting health facilities to verify and correct inaccurate and incomplete reports, but the final output was still not seen as a reliable representation of service delivery.

*“We currently face stock out problems because health workers have difficulties in measuring their monthly consumption. The ideal scenario would be if they could accurately estimate the monthly consumption so they order consequently.” (National Malaria Programme, Mali)*

To track programme performance, programme staff from EPI and ANC at the national level commonly reported using routine facility-based service delivery data. By contrast, in all four countries, it was far more common for malaria programme staff to identify surveys as the main data source for assessing national LLIN programme success.

*“We… do surveys or investigation to know how people sleep under a net.” (National Malaria Programme, Mali)*

*“Annually we are conducting the survey just to assess how much we have achieved in terms of coverage” (National Malaria Programme, Malawi)*

When asked directly, national malaria programme staff did confirm that they also use the routine LLIN distribution data collected at facilities to track the progress made in LLIN distribution.

At the facility level, staff rarely reported tracking their own progress using the service delivery data they collected. More often, health facility staff described collecting data for the purpose of reporting to the higher levels of the health system. The main exception to this was for non-LLIN programmes, when the facility was responsible for placing orders for a given commodity, such as vaccines or drugs.

## Discussion

In all four countries respondents at the facility level described an inconsistent supply of LLINs for continuous distribution, leading to stock-outs. As a result, the malaria programme in each country was unable to provide LLINs to the women and children attending ANC and EPI, when facility level stock-outs occurred. This unreliable supply of LLINs may also decrease the communities’ confidence in, and uptake of, ANC and EPI services (Fig. 1).

The main factor identified as contributing to stock-outs at the facility level was the facilities lack of involvement in supply decisions. Specifically, facilities did not participate in quantification exercises, were not given the authority to place regular orders for LLIN resupply, and did not have a system in place to report and remedy stock-outs when they occurred. A key issue underlying these constraints was that facility data was not deemed reliable by the higher service levels.

These findings suggest that, (a) facility-led resupply systems allowing local health facilities to request additional LLINs, (b) improved data collection and management during service delivery, and data use for quantification, and (c) the development of stock-out response systems, can improve the reliability and availability of LLIN stock at the facility level.

Malaria programme staff at the national and sub-national level expressed an interest in having facility level staff place orders for LLIN resupplies as needed. Despite this interest, a system of this nature had not been implemented in any of the countries at the time of this assessment. While the need for facility-led resupply has not been identified in previous LLIN research, it has been a finding in broader supply-chain studies. In 2001, a review of the Kenya medical supply agency (KEMSA) identified some similar areas for improvement including training, data management, and a feedback mechanism between facilities and national level programmes [21]. A study in Tanzania evaluated the impact of an integrated logistics system where facilities were given the responsibility of quantifying consumption and placing orders for supplies [22]. While there was an improvement in accountability of supplies in some commodity chains, the system did not fix the concerns of poor data and monitoring all together [22]. Both Rwanda and Malawi have recently been involved in a project to strengthen community health supply chains, focusing on antibiotics, ACT, ARTs, and ORS [23]. These evaluations both identified the need for products to be ordered based on facility need, through clear procedures [23]. They also noted the need for strong data management and use to ensure accurate information is available to inform management decisions [23].

These studies have recommended improved reliability of data, produced by facilities during service delivery, in conjunction with facility-led ordering system. This pairing helps national programmes to have confidence in orders placed at the facility level. Likewise, implementing partners and funding agencies have produced reports and guidelines on improving the supply chain for malaria programmes, which often recommend improving quality data management and use, especially for quantification [24–26]. Surveys were mentioned in all four countries as the primary tool for measuring LLIN programme performance. While surveys are the best way of evaluating LLIN use within communities, routine data collected at the time of service delivery are an untapped resource from which malaria programmes can benefit. Service delivery data has been collected, used and trusted by other programmes (such as vaccines), and could be an important source of information for malaria control programmes.

A better understanding of programme performance could be gained by ensuring LLIN distribution is routinely tracked as a key indicator and compared to ANC and EPI routine service delivery numbers. Similarly, an analysis of integrated community case management in 18 African countries also found that programmes would benefit from taking advantage of routine data for monitoring [27]. That analysis also recommended strengthened data use and response, and triangulation of routine data, as ways to improve the use of routine service delivery data [27].

Improved data collection and use can increase confidence in facilities’ ability to place orders, but it may not stop stock-outs from occurring all together. The availability of electronic health information systems did not improve stock-out response time in the three countries where they were present, though these systems were not available at the facility level at the time of the assessment. Likewise different frequencies of restock, from monthly to yearly, did not prevent stock-outs experienced in any of the four countries. As a result, a well-developed and standardized stock-out response system is necessary to ensure a continuous supply of LLINs at the facility level.

In all four countries, concerns were expressed about the lack of training specifically for the continuous distribution of LLINs. To develop a facility-led resupply system, and foster confidence in that system for staff at the higher levels, more emphasis on training could be made, especially around data collection and use. Trainings could be conducted as stand-alone sessions, as part of ANC or EPI trainings, or as part of broader malaria programme trainings.

Logistics challenges and stock-outs have been identified in LLIN research before. However, these concerns are often voiced in the discussion section of studies, while the main focus of the research is cost effectiveness, feasibility and/or scalability [12, 28–30]. In one study in Ghana, the transportation aspect of the supply chain, specifically, was seen as a contributor to stock-outs and supply shortages, which was not identified as a specific barrier in the four countries included in this study [16]. Beyond LLIN distribution, supply chain management has been a major focus of health systems research, for essential medicines, health commodities and vaccinations [23, 31–35].

The space nets take up is not trivial at any level of service delivery. In a study in Kenya, one district did not have a supply of nets because the bulk of the nets was underestimated, and so the space required to transport them was not available [10]. It is interesting to note that in this study, net-volume was mentioned as a burden when discussing storage, but was not brought up as a challenge when discussing logistics transportation in any of the four countries. While the reason for this is unknown—it may be because it is obvious, or alternatively because it is not actually a concern in these countries—it is worth noting that it was not mentioned.

There are several limitations of this study. Given the rapid nature of the project only 2–4 facilities were included, and 7–11 interviews were conducted with a total of 13–23 participants per country. As a result of the small numbers of facilities, it was not possible to include facilities that were both very strong and very weak in terms of service delivery. Therefore, in line with the intention of the research, the national malaria programmes each made an effort to select facilities that were “average performing” in terms of service delivery, and were “typical” in terms of access and distance to major cities. It was not possible to compare these facilities to those not chosen, so there is potential bias in the facility selection. While the findings represent the experiences in these facilities, the issues for facilities at the ends of the spectrum (very high or low performing) may be different. In two cases, originally selected facilities were replaced due to lack of access, as a result of flooding in Rwanda, and time constrains due to heavy work load in Malawi. Selection bias may have been introduced during the reselection process. Because of the small number of interviewees per country, this project may not have captured the total breadth of experiences in each country. On most topics, however, common experiences were recorded and reiterated across countries and interviews, suggesting that most important challenges were uncovered.

In one country, despite the intended protocol, a senior malaria programme staff member attended facility level interviews, which may have resulted in facility staff feeling less comfortable to speak freely. Likewise, in larger group interviews, especially in those at facilities where supervisors and general staff participated at the same time, there may have been a tendency for one person to lead in the answers. While questions were targeting different staff members, the responses were not always even. Experiences of divergent activities or criticism of policies or systems may have been voiced less frequently in these environments. Despite these limitations, valuable information was identified through interviews in all four countries, leading to a greater understanding of the operational challenges associated with the continuous distribution of LLINs.

## Conclusions

Each country is implementing a well-developed and planned LLIN distribution programme with clearly structured and presented policies and guidelines that have been communicated effectively at every level. While each country has created a unique and different policy and implementation plan, with differences in re-supply frequency and use of electronic databases, major cross cutting challenges can be seen as (1) facilities’ lack of involvement in the order and resupply process, and (2) the lack of structures in place to effectively and promptly respond to stock-outs at the facility level. These are undercut by a distrust in facility level data collected and reported to national programmes. These may also be challenges faced by other African countries implementing continuous LLIN distribution that were not included in this evaluation. Addressing these challenges has the potential to create a consistent and uninterrupted supply of LLINs, which is ultimately essential to make this programme truly a routine service.

Though it was not addressed in this research, the cost associated with improving these systems cannot be overlooked. Creating facility-led re-supply of LLINs will have financial implications which must be addressed in order to identify and implement cost-effective approaches and make any solution sustainable. One financial concern may be related to ensuring a “buffer stock” is maintained to resupply facilities when requested. As with all health commodities supplied continuously, the availability of stock, in country, to meet demand, is an essential part of routine service delivery. This would not require the purchase of additional LLINs, but rather, would require a consistent supply and storage of nets in country before they are needed. The storage facilities and stock already exist in country, but are not currently used as buffer stock, and thus not organized accordingly. Planning for and maintaining a buffer stock of LLINs will be an essential part of ensuring a truly continuous routine distribution of LLINs.

While the national level in all the countries did not see an immediate benefit to integrating the LLIN supply-chain with other health commodities, it may be an area that warrants further investigation. Facility level staff expressed an interest in an integrated system to improve service delivery. If implemented effectively, an integrated system may also provide cost savings due to shared services between programmes.

This study did identified specific operational barriers to the continuous distribution of LLINs leading to facility level stock-outs. Focusing on these barriers as priority areas for development and improvement can assist national programmes to identify specific solutions and tailored approaches that will work best in the unique context of each country.

## Additional files

10.1186/s12936-016-1184-y Facility Level Interview for NMCP, EPI and ANC.
10.1186/s12936-016-1184-y National Level Interview for NMCP, EPI and ANC.
