# Supplementary material for: Operational challenges to continuous LLIN distribution: a qualitative rapid assessment in four countries
Source: Malar J. 2016 Mar 1;15:131. doi: 10.1186/s12936-016-1184-y (PMC4774176; doi:10.1186/s12936-016-1184-y)
Supplement: Supplementary file 1 — 10.1186/s12936-016-1184-y Facility Level Interview for NMCP, EPI and ANC. [file 12936_2016_1184_MOESM1_ESM.docx]

**Facility Level Interview for NMCP, EPI and ANC**

(circle all that apply)

Do you work on: EPI ANC Malaria/LLINs

Level of work: National Regional Facility

Organization MOH Partner: ___________________________

General roles and responsibilities in your job:

| **Policy and Management:** |
| --- |

*POLICIES AND GUIDELINES*

- Do you use any policies or guidelines for your work each day? (EPI schedule, ANC services, integrated services, broad LLIN delivery, etc)
- Are there SOPs in place for service delivery?
- Are there guidelines, job aides, or SOPs for your work that specifically mention integrated services and/or continuous LLIN distribution?

*PROGRAMME MANAGEMENT*

- Who is responsible for programme management and implementation at this facility? Who oversees the delivery of this programme?
- Are any persons specifically responsible for integration?
- How is success defined and measured for the programme?
- Is coverage used as a measure, and how is it defined?

What policy and management factors, if any, contribute to the success of this programme? (General programme success and specific integration success)

What policy and management factors, if any, hinder the success of this programme? (General programme success and specific integration success)

| **Programme Implementation and Human Resources:** |
| --- |

*PROGRAMME*

- Can you explain how service delivery works?
- At which point in ANC and EPI are nets distributed? Are there other ways that nets are distributed?
- Who is responsible for providing each service?
- Is there a cost associated with any of the services provided?
- Do any incentives exist for performance?
- Is there enough time to complete all the tasks during ANC and EPI visits?
- Is there a difference between LLINs for ANC, EPI and campaigns?
- Are there times when someone does not get an LLIN who should receive one? Can you explain how that happens?
- Are there times when someone receives an LLIN who is not technically eligible? Can you explain how that happens?

*HUMAN RESOURCES*

- What kind of training, if any, did you receive for each programme or service that you provide? (stock management, service delivery, reporting, quantification, etc)
- Is there on-site supervision for any of the programme?

What programme delivery and/or human resource factors, if any, contribute to the success of this programme?

What programme delivery and/or human resource factors, if any, hinder the success of this programme?

| **Logistics:** |
| --- |

*SUPPLY QUANTIFICATION AND STOCK MANAGEMENT*

- What are all the commodities and supplies associated with your programme?
- How is supply quantification and expected need calculated?
- Who reports on stock levels at this facility? Where do reports go?
- How are more supplies ordered and delivered to this facility?
- What kind of stock-management system is in place?
- How often are new shipments of supplies received at this facility?
- Is there any separation of supplies for campaigns compared to those for routine health service distribution? (primarily for LLINs, but also for vaccine supply management)

*SUPPLY DISTRIBUTION*

- Where do the supplies come from for this facility?
- Do all health supplies come from the same place?

What logistics system factors, if any, contribute to the success of this programme?

What logistics system factors, if any, hinder the success of this programme?

| **Data Collection, Management and Use:** |
| --- |

*PROGRAMME DATA*

- What data are recorded at the facility level during service delivery?
- How are those data collated?
- Where do data reports go?
- How is service delivery data used at this facility? (Do you aggregate the data? Do you measure against targets?)
- What kind of feedback do you receive based on reports?
- Do you monitor the programme here at the facility? Do you use coverage data for programme monitoring? How is it measured?

*LOGISTICS DATA*

- What data are collected and kept for stock management?
- How are stock-management data used?
- What kind of stock-level data and reports are completed here?
- What kind of feedback exists for stock management?
- What tools (if any) exist for quantification of stock needs?

What data collection, management and use factors, if any, contribute to the success of this programme?

What data collection, management and use factors, if any, hinder the success of this programme?
