# Supplementary material for: Operational challenges to continuous LLIN distribution: a qualitative rapid assessment in four countries
Source: Malar J. 2016 Mar 1;15:131. doi: 10.1186/s12936-016-1184-y (PMC4774176; doi:10.1186/s12936-016-1184-y)
Supplement: Supplementary file 2 — 10.1186/s12936-016-1184-y National Level Interview for NMCP, EPI and ANC. [file 12936_2016_1184_MOESM2_ESM.docx]

**National Level Interview for NMCP, EPI and ANC**

(circle all that apply)

Do you work on: EPI ANC Malaria/LLINs

Level of work: National Regional Facility

Organization MOH Partner: ___________________________

**General roles and responsibilities in your job:**

| **Policy and Management:** |
| --- |

*POLICIES AND GUIDELINES*

- What policies and guidelines exist for the implementation and service delivery of your programme? (EPI schedule, ANC services, integrated services, broad LLIN delivery, etc)
- What policies exist specific to integrated services and/or continuous LLIN distribution?
- How do you understand these policies to be implemented?
- Are there SOPs in place for service delivery?

*PROGRAMME MANAGEMENT*

- Who is responsible for programme management and implementation at each level?
- Are any persons specifically responsible for integration?
- How are integrated programmes managed and coordinated (at each level)?
- Is there specific cross-department coordination between Malaria, EPI, and ANC/MCH at the National Level?
- How is success defined and measured for the programme?
- Is coverage used as a measure, and how is it defined?

What policy and management factors, if any, contribute to the success of this programme? (General programme success and specific integration success)

What policy and management factors, if any, hinder the success of this programme? (General programme success and specific integration success)

| **Logistics:** |
| --- |

*SUPPLY QUANTIFICATION AND STOCK MANAGEMENT*

- What are all the commodities and supplies associated with your programme?
- How is national, regional, and facility need and quantification performed?
- What kind of stock-management system is in place?
- How often are new shipments of supplies received in country? When, in the last year were shipments received?
- Is there any separation of supplies for campaigns compared to those for routine health service distribution? (primarily for LLINs, but also for vaccine supply management)

*SUPPLY DISTRIBUTION*

- How is the distribution of commodities and supplies set up?
- How many stops/hubs are there between National stores and health facilities or the point of distribution/use?
- Are these commodities distributed in the same supply chain as other health commodities, or using a different supply chain?

*DISTRIBUTION MONITORING AND REPORTING*

- Who reports on stock levels at facility, regional, and national levels?
- How are more supplies ordered and delivered to facilities?
- What kind of stock management systems are in use?

What logistics system factors, if any, contribute to the success of this programme?

What logistics system factors, if any, hinder the success of this programme?

| **Programme Implementation and Human Resources:** |
| --- |

*PROGRAMME*

- Can you explain how service delivery works?
- At which point in ANC and EPI are nets distributed?
- Who is responsible for providing each service?
- Who is responsible for integrated service management?
- Is there a cost associated with any of the services provided?
- Do any incentives exist for performance?

*HUMAN RESOURCES*

- What kind of training, if any, is given to health facility staff?
- How is distribution monitored?
- Is there on-site supervision for any of the programme?

What programme delivery and/or human resource factors, if any, contribute to the success of this programme?

What programme delivery and/or human resource factors, if any, hinder the success of this programme?

| **Data Collection, Management and Use:** |
| --- |

*PROGRAMME DATA*

- What data are recorded at the facility level during service delivery?
- How are those data collated?
- Where do data reports go?
- How is service delivery data used at the facility, regional, and national level?
- What kind of feedback is given to regions and facilities in response to service delivery reports?
- What non-routine data collection methods/surveys does the programme rely on for performance measures?
- Are coverage data used for programme monitoring? How is it measured?

*LOGISTICS DATA*

- What data are collected and kept for stock management?
- How are stock-management data used?
- What kind of stock-level data and reports are completed at each level?
- What kind of feedback exists for stock management?
- What tools (if any) exist for quantification of stock needs?

What data collection, management and use factors, if any, contribute to the success of this programme?

What data collection, management and use factors, if any, hinder the success of this programme?
